# Inhibition of smooth muscle cell death by Angiotensin 1-7 protects against abdominal aortic aneurysm

**DOI:** 10.1042/BSR20230718

**Published:** 2023-11-30

**Authors:** Anshul S. Jadli, Karina P. Gomes, Noura N. Ballasy, Tishani Methsala Wijesuriya, Darrell Belke, Paul W.M. Fedak, Vaibhav B. Patel

**Affiliations:** 1 Department of Physiology and Pharmacology, Cumming School of Medicine, University of Calgary, Calgary, AB, Canada; 2 Libin Cardiovascular Institute, University of Calgary, Calgary, AB, Canada; 3 Section of Cardiac Surgery, Department of Cardiac Sciences, Cumming School of Medicine, University of Calgary, Calgary, AB, Canada

**Keywords:** Abdominal aortic aneurysm, Angiotensin 1-7, Mitochondrial fission, Renin-angiotensin system, Smooth muscle cells

## Abstract

Abdominal aortic aneurysm (AAA) represents a debilitating vascular disease characterized by aortic dilatation and wall rupture if it remains untreated. We aimed to determine the effects of Ang 1-7 in a murine model of AAA and to investigate the molecular mechanisms involved. Eight- to 10-week-old apolipoprotein E-deficient mice (ApoEKO) were infused with Ang II (1.44 mg/kg/day, s.c.) and treated with Ang 1-7 (0.576 mg/kg/day, i.p.). Echocardiographic and histological analyses showed abdominal aortic dilatation and extracellular matrix remodeling in Ang II-infused mice. Treatment with Ang 1-7 led to suppression of Ang II-induced aortic dilatation in the abdominal aorta. The immunofluorescence imaging exhibited reduced smooth muscle cell (SMC) density in the abdominal aorta. The abdominal aortic SMCs from ApoEKO mice exhibited markedly increased apoptosis in response to Ang II. Ang 1-7 attenuated cell death, as evident by increased SMC density in the aorta and reduced annexin V/propidium iodide-positive cells in flow cytometric analysis. Gene expression analysis for contractile and synthetic phenotypes of abdominal SMCs showed preservation of contractile phenotype by Ang 1-7 treatment. Molecular analyses identified increased mitochondrial fission, elevated cellular and mitochondrial reactive oxygen species (ROS) levels, and apoptosis-associated proteins, including cytochrome *c*, in Ang II-treated aortic SMCs. Ang 1-7 mitigated Ang II-induced mitochondrial fission, ROS generation, and levels of pro-apoptotic proteins, resulting in decreased cell death of aortic SMCs. These results highlight a critical vasculo-protective role of Ang 1-7 in a degenerative aortic disease; increased Ang 1-7 activity may provide a promising therapeutic strategy against the progression of AAA.

## Introduction

An abdominal aortic aneurysm is characterized by localized, irreversible dilatation of the aorta in the abdominal region >50% of the normal aorta diameter [1]. AAA predominantly occurs in the infrarenal region of the aorta, proximal to its bifurcation into common iliac arteries [2,3]. Age is a significant risk factor for the development of AAA, which affects approximately 5% of the elderly population, primarily males, and causes significant mortality in Western countries. Clinical progression of AAA involves the weakening of the aortic wall, resulting in progressive dilatation and, eventually, wall rupture, a catastrophic event associated with a mortality rate of 80% [2]. Pathologically, AAA is associated with aortic smooth muscle cell (SMC) apoptosis, inflammation, extracellular matrix (ECM) remodeling, i.e., disruption of elastin fibers and collagen deposition, upregulation of proteolytic pathways, and oxidative stress [1]. The multifactorial nature and absence of precise delineation of pathological mechanisms involved in AAA development have resulted in the absence of effective therapeutic intervention.

The renin–angiotensin system (RAS) is a hormonal system primarily involved in the physiological regulation of blood pressure and vascular tone. Overactivation of RAS predisposes the cardiovascular system to maladaptive and pathological perturbations [4,5]. Angiotensin II (Ang II) is an effector peptide of RAS that plays a pivotal role in vascular inflammation, endothelial dysfunction, and fibrosis in various cardiovascular diseases [6]. Ang II exerts its physiological and pathological effects primarily through the activation of the Ang II type 1 (AT1R) receptor and partly through the Ang II type 2 (AT2R) receptor [7]. Multiple mechanisms have been proposed for the contribution of Ang II to the development of AAA. These comprise infiltration of immune cells and consequent inflammatory responses, degradation of extracellular matrix proteins via activation of proteases, vascular oxidative stress, and aortic smooth muscle cell death [4,8]. Several investigators assessed the effect of medications in suppressing aneurysm development in *in vitro* experiments and preclinical models [9–11]. Various studies reported statins, inhibitors of Angiotensin II production and signaling (AT1R blockers and angiotensin-converting enzyme (ACE) inhibitors), matrix metalloproteinases (MMP) inhibitors, cyclooxygenase, and macrolides inhibitors as potential treatment options. These studies emphasized their ability to reduce inflammation, ECM remodeling, and oxidative stress associated with the pathogenesis of AAA. However, clinical trials investigating the therapeutic potential of these medications in suppressing aortic dilatation demonstrated inconsistent results with significant side effects [12,13]. The translational gap between preclinical accomplishments and clinical trial failures suggests a partial representation of complex human pathology by animal models and the need to investigate further elusive crucial pathogenic mediators of AAA development [14]. Since no drug has demonstrated clinical efficiency in limiting AAA progression or rupture, surgical interventions remain an active treatment option. AAA repair by either open or minimally invasive surgery is indicated for symptomatic or ruptured AAA of any size or large, asymptomatic AAA [15]. While open surgeries are associated with significant perioperative mortality, long-term effects of endovascular repairs include late AAA rupture and endoleak-the persistent perfusion of the AAA sac from side branches of the aorta or the leaks around the stent [16]. The risk associated with aortic surgery and AAA repairs has shifted focus toward identifying novel pharmacological interventions to slow or reverse, aneurysmal growth or delay the need for surgery to manage large AAAs.

Angiotensin 1-7 (Ang 1-7) is a biologically active heptapeptide cleaved predominantly from Ang II by the action of ACE2, a recently identified homolog of ACE. Ang 1-7 has been shown to prevent adverse cardiovascular effects of Ang II/AT1R activation [17], such as cardiac arrhythmia, heart failure, renal diseases, and hypertension [18,19]. By stimulating the activity of vasodilator autocoids and nitric oxide (NO), Ang 1-7 counteracts the vasoconstrictive effects of Ang II [20]. Ang 1-7 exhibited a protective role in systemic hypertension, oxidative stress, and vascular disorders associated with vascular remodeling [21,22]. Though a recent study demonstrated Ang 1-7-mediated anti-inflammatory and anti-apoptotic effects in the aortic aneurysm [23], the precise mechanism of Ang 1-7-mediated antagonism of Ang II is still being investigated as the Ang 1-7/Mas receptor axis has been shown to counteract Ang II/AT1R axis in cardiovascular diseases [24,25]. In the present study, we assessed the ability of Ang 1-7 to attenuate the development and progression of AAA in a murine model and investigated the molecular mechanisms involved.

## Materials and methods

### Experimental animals

*ApoE*-deficient mice (ApoEKO; C57BL/6 background) were purchased from the Jackson Laboratory (Bar Harbor, Me) and bred at the Health Sciences Animal Resource Centre of the University of Calgary. Mice were housed in pathogen‐free conditions and had access to sterilized food and water *ad libitium*. All the animal experiments were performed at the Department of Physiology and Pharmacology of Cumming School of Medicine at the University of Calgary. Alzet osmotic pumps (Model 1004, Durect Corp.) were implanted subcutaneously in 8- to 10-week-old male mice to deliver Ang II (1.5 mg/kg/day) or saline (control) for 4 weeks [26]. A subgroup of ApoEKO mice receiving Ang II infusion was also implanted with osmotic pumps intraperitoneally to deliver Ang 1-7 (0.5 mg/kg/day). The mice were anesthetized using isoflurane (5% induction, followed by 2% maintenance in 500 ml/min oxygen flow). The mice used in the study were killed by carbon dioxide asphyxiation. The Institutional Animal Ethics Committee of the University of Calgary approved the study (AC21-0029). All experiments were conducted as per the guidelines of the University of Calgary Animal Care and Use Committee and the Canadian Council of Animal Care.

### Echocardiography

Ultrasonic images of the aorta were obtained using a Vevo 3100 high-resolution imaging system equipped with a real-time micro visualization scan head (MX550D, VisualSonics). Briefly, mice were anesthetized with 2% isoflurane mixed with O_2_, as previously described [26]. The diameters of the abdominal aorta were measured using M-mode images. The maximum and minimum aortic lumen diameters, corresponding to cardiac systole and diastole, respectively (monitored by simultaneous ECG recordings), were measured and used to calculate the aortic expansion index [(Systolic aortic diameter − Diastolic aortic diameter)/Systolic diameter × 100]. The aortic distensibility was calculated using B-mode and EKV images using the Vevo Vasc analysis package of the Vevo LAB software (VisualSonics).

### Isolation and culture of aortic smooth muscle cells

SMCs were isolated from abdominal aortas harvested from 8- to 10-week-old ApoEKO mice and were cultured as previously described [26]. Aortas were harvested in aseptic conditions, and periaortic adipose tissues were cleaned. Abdominal aortas were incubated with a digestive enzyme solution containing 1 mg/ml collagenase II (#LS004174, Worthington Biochemical) and 1 mg/ml elastase (#LS002292, Worthington Biochemical) at 37°C in a 5% CO_2_ incubator for 10 min. The adventitial and inner layers of the aorta were then removed under a surgical microscope. Medial layers of the aortas were cut into small pieces and incubated with the digestive enzyme solution for 90 mins at 37°C in a 5% CO_2_ incubator. After digestion, cells were washed and cultured in a sterile DMEM/F12 cell culture medium (#11330057, Gibco) containing 20% fetal bovine serum (FBS, #26140079, Gibco) supplemented with 1% penicillin/streptomycin (#P0781, Sigma-Aldrich). Cells were passaged upon reaching 90–100% confluency. Abdominal aortic SMCs were serum‐deprived for 24 h by incubation in DMEM/F12 medium with 1% fetal bovine serum and penicillin/streptomycin. Cells were challenged with Ang II (100 nM; #05-23-0101, Millipore Sigma) in DMEM/F12 for 24 h. In a treatment group, Ang 1-7 (100 nM) (#H-1715.0025, Bachem) was added to the SMC culture 30 min before Ang II exposure. A subgroup of SMCs without Ang II and Ang 1-7 treatments served as controls.

### Histological analysis and immunofluorescence staining

After 4 weeks of Ang II (±Ang 1-7) infusion, mice from saline (*n*=7), Ang II (*n*=4) and Ang II+Ang 1-7 (*n*=4) groups were perfuse‐fixed using 10% buffered formalin or saline at a pressure equivalent to 80 mmHg, allowing the blood vessels to be fixed in their native state, as we have previously described [27]. Subsequently, aortas were collected and fixed in 10% buffered formalin for 48 h and later embedded in paraffin blocks. Five μm thick sections were used for the structural histological assessment using hematoxylin and eosin (H&E), Gomori trichrome, and Verhoeff-Van Gieson (VVG) staining as previously described [26,27]. Picro-Sirius red (PSR) staining was used to evaluate collagen levels and adventitial fibrosis, as previously described [28]. PSR-staining positive (collagen) area in the abdominal aorta was imaged using a laser scanning confocal microscope (Leica SP8) and quantified using the Fiji/ImageJ (version 1.52i) software. As previously described, 5 μm thick sections were used for the immunofluorescence staining [26,27]. After deparaffinization, sections were immersed in EDTA-citrate buffer (pH 6.2) at 95°C for 20 minperform antigen retrieval. After that, sections were washed with PBS and permeabilized with Triton X-100 (0.1%), followed by blocking with bovine serum albumin (BSA; 1.5%). Sections were incubated with primary antibody against α-smooth muscle actin (α-SMA, 1:500, #ab32575, Abcam) overnight at 4°C, followed by Alexa Fluor 488-conjugated Goat anti-Rabbit secondary antibody (1:400, #A11034, Invitrogen) at room temperature for 1 h. Sections were mounted and counterstained using Prolong Gold mounting medium with DAPI (#P36935, Invitrogen). Immunostained aorta sections were imaged using a line scanning confocal microscope (Leica SP8) and analyzed using the Fiji/ImageJ (version 1.52i) software.

### Annexin V staining and cell death assay

Apoptotic cell death was evaluated using the Annexin V/propidium iodide staining, and flow cytometric analysis as previously described [26]. Control and Ang II (±Ang 1-7)-treated abdominal aortic SMCs were trypsinized to collect adherent cell monolayers and floating cells and centrifuged at 1500 ***g*** for 5 min. The cell pellets were washed thrice in PBS and re-suspended in annexin binding buffer at 1 ×10^6^ cells/ml. The cells were then incubated with FITC-conjugated Annexin V antibody (#640945, Biolegend) and propidium iodide (1 μg/ml; #LS00699050, Invitrogen) at room temperature for 15 min. After the incubation, the annexin‐binding buffer was added. Samples were analyzed within 1 h using Attune NxT flow cytometer (ThermoFisher Scientific, MA, U.S.A.) at 488 nm excitation and 530 and 575 nm emission wavelengths. Quantitative analysis of flow cytometric data was performed using Invitrogen™ Attune™ NxT Software (version 2.6, ThermoFisher Scientific, MA, U.S.A.).

### Quantitative real-time PCR

The gene expression profile for contractile and synthetic genes was performed as described previously [27,29]. Following TaqMan probes for quantitative PCR were used to measure the expression of specified genes: *Acta2* (Mm.PT.58.16320644), *Myh11* (Mm.PT.58.9236105), *Il-6* (Mm.PT.58.10005566), *Mmp2* (Mm00439498_m1), *Mmp9* (Mm00442991_m1), *Col1a1* (Mm00801666_g1), *Col3a1* (Mm00802331_m1), and *18S rRNA* (Hs.PT.39a.22214856.g). The gene expression was quantified by the 2^−ΔΔCT^ method using *18S* rRNA as endogenous control. Expression analysis of the reported genes was performed by TaqMan Real-time PCR using the QuantStudio™5 system (Thermo Fisher Scientific, MA, U.S.A.). Data were analyzed using QuantStudio™ design and analysis software version 1.4.3. All samples were analyzed in triplicates in 384 well plates.

### MitoTracker Red staining

The mitochondrial structure was assessed using MitoTracker Red CMXRos (#M7512, Invitrogen) staining and confocal imaging. Briefly, control or Ang II (±Ang 1-7)-treated abdominal aortic SMCs were incubated with 100 nM Mitotracker Red CMXRos for 30 min at 37°C in a CO_2_ incubator. MitoTracker Red-labelled cells were washed with PBS and fixed using 4% paraformaldehyde. Cells were then mounted and counterstained using Prolong Gold mounting medium with DAPI (#P36935, Invitrogen) and imaged using a line-scanning confocal microscope (Leica SP8). Fiji/ImageJ (version 1.52i) and Mitochondrial Network Analysis (MiNA) plugin were used to assess the mitochondrial structure as previously described [30].

### Cellular reactive oxygen species (ROS) analysis

Cellular ROS generation was visualized using the previously described dihydroethidium (DHE) staining [26]. Control and Ang II (±Ang 1-7)-treated abdominal aortic SMCs were initially incubated with DHE (10 μM; #D11347, Invitrogen) at 37°C for 30 min in a CO_2_ incubator. The cells were imaged using a line-scanning confocal microscope (Leica SP8). Quantitative measurement of DHE fluorescence intensity was carried out using the Fiji/ImageJ (version 1.52i) software.

### Mitochondrial ROS analysis

Mitochondrial ROS generation was assessed using MitoSOX Red mitochondrial superoxide indicator (#M36008, Invitrogen). Control and Ang II (±Ang 1-7)-treated abdominal aortic SMCs were trypsinized to collect adherent and detached cells and were centrifuged at 1500 ***g*** for 5 min. Cell pellets were washed and resuspended in HBSS/Ca^2+^ buffer at 0.5 ×10^6^ cells/ml. Cells were then incubated with 1 µM MitoSOX Red at 37°C for 10 min in the dark. After the incubation, cells were washed and resuspended in HBSS/Ca^2+^ buffer for flow cytometric analysis using Attune NxT flow cytometer (ThermoFisher Scientific, MA, USA) at excitation and emission wavelengths of 510/580 nm. The Invitrogen™ Attune™ NxT Software (version 2.6, ThermoFisher Scientific, MA, U.S.A.) was used to analyze the MitoSOX fluorescence intensity, representing the mitochondrial ROS levels.

### Apoptosis array

A proteome profiler mouse apoptosis array kit (#ARY031, R&D Systems, MN, U.S.A.) was used to assess expression levels of selected apoptosis-related proteins. The kit contained capture antibodies, specific for 21 apoptosis-related proteins, and control antibodies spotted in duplicate on nitrocellulose membrane. Membranes were blocked for 1 h at room temperature. Whole-cell lysates from control or Ang II (±Ang 1-7)-treated abdominal aortic SMCs were incubated overnight with the membranes at 4°C. The membranes were washed to remove unbound proteins and incubated with a cocktail of biotinylated detection antibodies for 60 min at room temperature. Streptavidin-HRP and chemiluminescent detection reagents were applied for 30 min at room temperature. Chemiluminescent images were captured using the Invitrogen iBright FL1500 imaging system (ThermoFisher Scientific). A signal produced at each capture spot corresponding to the amount of protein bound to the antibody was analyzed using iBright analysis software (Invitrogen). It was presented as a fold change to corresponding control spots.

### Statistical analysis

All data are shown as mean ± SEM. All statistical analyses were performed using GraphPad Prism v9 (San Diego, CA, U.S.A.). The data between Control, Ang II, and Ang II+Ang 1-7 groups were compared using one-way ANOVA followed by Tukey’s multiple comparisons post hoc analysis. The Kaplan–Meier survival analysis with Log-rank (Mantel-Cox) test used for the interpretation of survival proportions. *P*-value <0.05 was considered statistically significant.

## Results

### Ang 1-7 alleviates the development of AAA in a murine model

**Figure 1:**
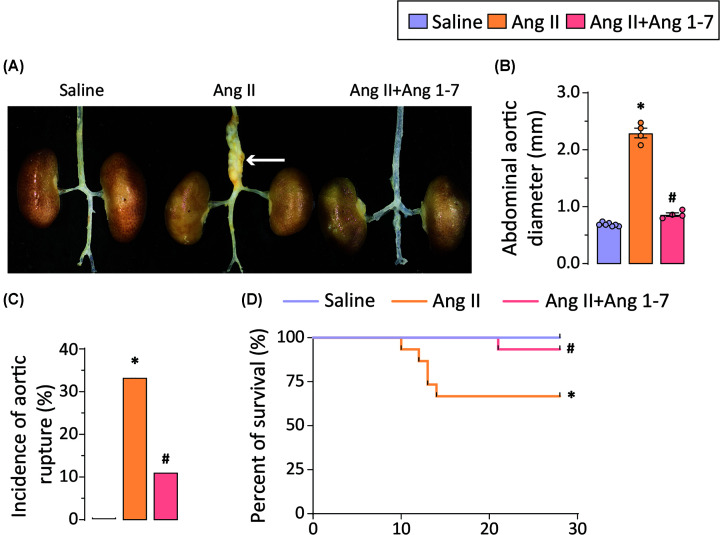
Ang 1-7 abrogates the development of Ang II-induced abdominal aortic aneurysm and associated mortality in ApoE knockout mice (**A**) Representative photographs of the aorta showing macroscopic features of aortic dilatation and (**B**) quantification of abdominal aortic diameter in the ApoEKO mice in response to saline (*n*=25), Ang II (*n*=7) and Ang 1-7 (*n*=8) for 4 weeks. (**C**) The administration of Ang II leads to a higher incidence of abdominal aortic rupture (4/12 = 33.33%; 1: unknown cause; **D**), resulting in significantly increased mortality (*n*=5) in ApoEKO mice compared to the saline group (0/25 = 0.00%). (**C,D**) Ang 1-7 prevents aortic dilatation and reduces the incidence of aortic rupture (1/9 = 11.11%), resulting in improved survival. The arrow points to the aneurysmal region. Each data point on graph represents biological replicate. *P<0.05 compared with the saline group; #*P*<0.05 compared with the Ang II group using one-way ANOVA.

Chronic subcutaneous infusion of Ang II in ApoEKO mice has been well characterized to develop AAA [31,32]. Consistently, we observed increased aortic dilatation and aneurysm formation in ApoEKO mice receiving 4 weeks of Ang II infusion (Figure 1A,B). Increased incidences of aortic rupture and reduced survival were observed in response to Ang II infusion (Figure 1C,D). The increased mortality in Ang II infused in ApoEKO mice was due to the abdominal aortic rupture. Effects of Ang 1-7, a vasoprotective peptide, were evaluated on the murine model of AAA [26]. Intraperitoneal administration of Ang 1-7 mitigated abdominal aortic dilatation and aneurysm formation, as exhibited by reduced aortic diameters compared to Ang II-infusion (Figure 1A,B). Moreover, Ang 1-7 treatment also resulted in decreased %incidences of aortic rupture and associated mortality (Figure 1C,D), suggestive of potent vasculoprotective effects of Ang 1-7 in AAA.

**Figure 2:**
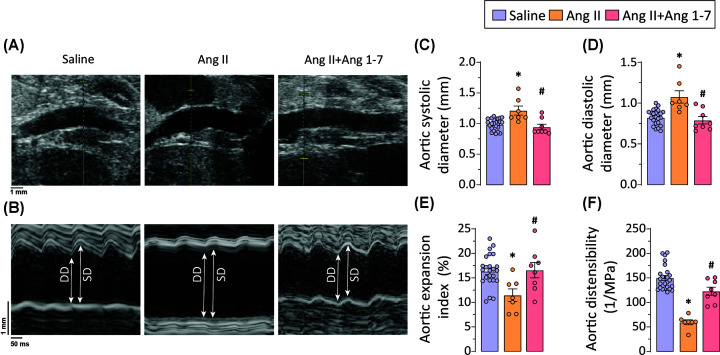
Ang 1-7 attenuates Ang II-induced structural and functional alterations in abdominal aorta in ApoE knockout mice Representative (**A**) B-mode and (**B**) M-mode echocardiography images of the abdominal aorta and quantification of abdominal aortic (**C**) systolic and (**D**) diastolic diameters showing increased aortic dilatation in Ang II-infused ApoEKO mice; Chronic Ang 1-7 administration markedly reduced (A–D) the aortic dilatation. Quantification of (**E**) aortic expansion index and (**F**) aortic distensibility exhibits markedly reduced aortic wall elasticity and distensibility in response to Ang II-infusion in ApoEKO mice; chronic Ang 1-7 administration preserved the aortic (E) expansion index and (F) distensibility. Each data point on graph represents biological replicate. *P<0.05 compared with the saline group; #*P*<0.05 compared with the Ang II group using one-way ANOVA. DD: diastolic diameter, SD: systolic diameter.

Transthoracic murine echocardiography evaluated the structural and functional changes in response to 4 weeks of Ang II (±Ang 1-7) infusion. Chronic Ang II infusion increased aortic lumen diameter at systole and diastole (Figure 2A–D), exhibiting a key defining feature observed in patients with abdominal aortic aneurysms. Chronic Ang II administration resulted in decreased abdominal aortic expansion index (Figure 2E), an indicator of biomechanical elasticity and recoil property of the aorta. Moreover, structural changes in Ang II-infused aorta were corroborated by assessing aortic distensibility, a reliable and sensitive measure of aortic wall property. Aortic wall distensibility is described as the ability of the aorta to expand during systole. It represents a measure to assess the biomechanical property of the aorta that characterizes aortic stiffness and the risk of aortic wall rupture [33]. The high-frequency ECG-gated Kilohertz Visualization (EKV)-mode of echocardiography was used to evaluate aortic distensibility in the abdominal aorta, which showed markedly reduced distensibility in response to Ang II infusion (Figure 2F). Ang 1-7 administration attenuated Ang II-induced aortic dilatation (Figure 2A–D) along with preserved aortic expansion index (Figure 2E) as well as aortic distensibility (Figure 2F), implying decreased adverse vascular remodeling.

### Ang 1-7 attenuates pathological aortic remodeling in a murine model of AAA

**Figure 3:**
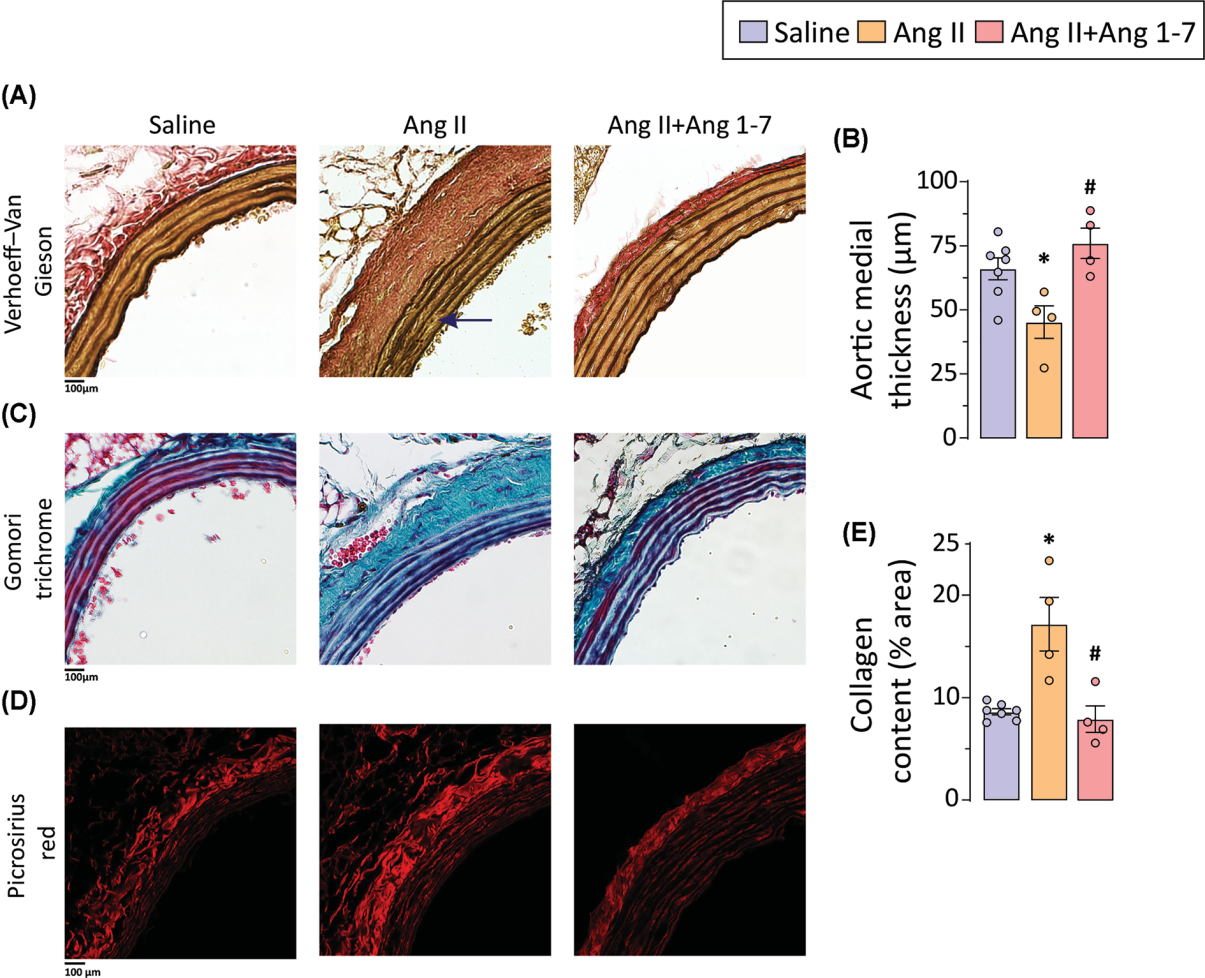
Ang 1-7 suppresses Ang II-induced adverse aortic remodeling in the abdominal aorta Representative images of (**A**) Verhoeff-Van Gieson (elastin) staining and brightfield microscopy and (**B**) quantification of aortic media thickness show significantly decreased aortic medial thickness and increased medial degeneration with elastin breaks in the abdominal aorta of ApoEKO mice in response to Ang II-infusion; chronic Ang 1-7 administration reduced medial degeneration and preserved abdominal aortic medial thickness. Representative images of (**C**) Gomori trichrome staining and brightfield microscopy and (**D**) picrosirius red (collagen) staining and confocal microscopy along with (**E**) quantification of collagen content indicate increased ECM remodeling in response to Ang II administration and (C–E) abridged adventitial fibrosis resulting from chronic Ang 1-7 administration. Each data point on graph represents biological replicate. *P<0.05 compared with saline group; #*P*<0.05 compared with Ang II group using one-way ANOVA.

Structural changes in the aortic wall usually accompany abdominal aortic dilatation and aortic lumen dilatation. Histological analysis using Verhoeff-Van Gieson (VVG) staining revealed disorganized and disrupted elastin fibers in the abdominal aorta of Ang II-infused mice compared to controls (Figure 3A). Moreover, Ang II infusion also reduced aortic medial thickness (Figure 3B), correlating with medial degeneration observed in patients with AAA. ECM remodeling, including excessive adventitial collagen deposition, has been implicated in the onset and progression of AAA [34]. Gomori trichrome and picrosirius red staining was performed to determine the collagen levels. Chronic Ang II infusion increased collagen deposition in the adventitial layers of the abdominal aorta, indicating manifestation of adventitial fibrosis and ECM remodeling (Figure 3C–E) in the murine model of AAA. Notably, chronic administration of Ang 1-7 preserved the structural integrity of elastin fibers (Figure 3A), alleviated medial degeneration resulting in preserved aortic medial thickness (Figure 3B), and reduced adventitial fibrosis (Figure 3C–E).

### Ang 1-7 inhibits Ang II-mediated apoptotic loss and phenotypic switching of abdominal aortic SMCs

**Figure 4:**
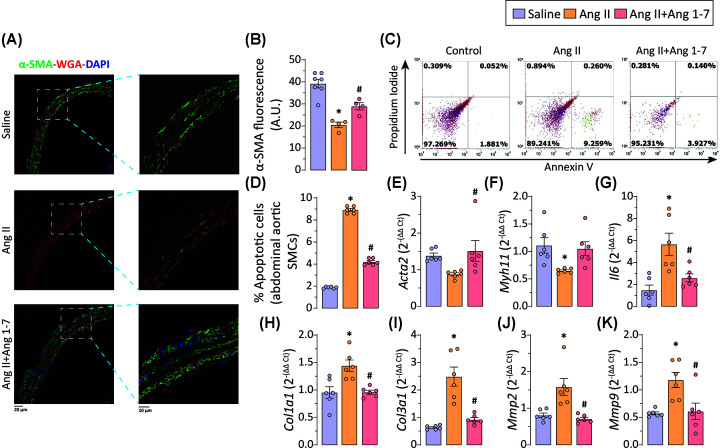
Ang 1-7 attenuates Ang II-induced apoptosis and phenotypic switching in abdominal aortic SMCs Representative (**A**) immunofluorescence and confocal microscopy images of α-SMA (green) and wheat-germ agglutinin (red) staining of the abdominal aorta and (**B**) quantification of α-SMA-immunoreactivity exhibit reduced α-SMA fluorescence suggesting loss of VSMCs density in aortic media by Ang II administration and (A,B) preservation of α-SMA levels by Ang 1-7 administration in ApoEKO mice. (**C**) Flow cytometric analysis of annexin V/propidium iodide–stained vascular smooth muscle cells (VSMCs) and (**D**) quantification of % apoptotic cells showing increased apoptosis in the aortic VSMCs in response to Ang II and suppressed by Ang 1-7 stimulation. TaqMan-qPCR based mRNA expression analyses of α-smooth muscle actin (*Acta2*; **E**), myosin heavy chain 11 (*Myh11*; **F**), and interleukin-6 (*Il-6*; **G**) shows decrease expression of contractile genes and increased expression of the inflammatory gene in Ang II-treated abdominal SMCs. mRNA expression analyses of collagen I (*Col1a1*; **H**), collagen III (*Col3a1*; **I**), matrix metalloproteinase 2 (*Mmp2*; **J**), and matrix metalloproteinase 9 (*Mmp9*; **K**), indicate pathological polarization of abdominal aortic SMCs to a ‘synthetic’ phenotype in response to Ang II. Ang 1-7 treatment preserved the contractile phenotype of abdominal aortic SMCs (*n* = 6 in each group) (E**–**K). Each data point on graph represents biological replicate. *P<0.05 compared with saline group; #*P*<0.05 compared with Ang II group using one-way ANOVA.

Loss of SMC density in the medial layer of the aorta has been recognized as one of the major factors driving the onset and progression of AAA [26,35–37]. Confocal images of immunostained sections of the abdominal aorta showed decreased α-smooth muscle actin (α-SMA)-positive cells in the medial layer, indicating loss of medial SMC density in chronically Ang II-infused ApoEKO mice (Figure 4A,B). As the *in vivo* experiments suggested loss of aortic SMCs density in Ang II-infused aorta, *in vitro* investigations using isolated abdominal aortic SMCs were performed to delineate the underlying mechanism. Ang II has previously been shown to exert a proapoptotic effect on aortic SMCs, facilitating the development of various cardiovascular diseases [26,38]. Apoptotic loss of aortic SMCs and consequent medial degeneration have been identified as crucial pathological observations in patients with AAA [26,35–37]. *In vitro*, increased apoptosis was observed in abdominal aortic SMCs acutely challenged with Ang II. Flow cytometric analysis indicates increased Annexin V^+^/Propidium Iodide^−^ cells in the Ang II treatment group (Figure 4C,D). It is widely acknowledged that the phenotypic switching of VSMCs is a crucial factor in the development and progression of AAA [39,40]. The transformation of VSMC phenotype from contractile to synthetic triggered by Ang II caused vascular remodeling and inflammation, leading to the development of an AAA [41,42]. We investigated Ang 1-7-mediated effect on abdominal aortic SMCs phenotypic switching using quantitative mRNA expression analysis. Gene expression analysis revealed reduced expression of contractile genes, including *Acta2 and Myh11* (Figure 4E,F), in Ang II-treated abdominal aortic SMCs. Ang II treatment of the abdominal aortic SMCs also resulted in increased mRNA expressions of *Il-6, Mmp2, Mmp9, Col1a1*, and *Col3a1* (Figure 4G–K), signifying their polarization toward inflammatory and synthetic phenotypes. Co-treatment with Ang 1-7 preserved the contractile phenotype of abdominal aortic SMCs, as evident by preserved mRNA expressions of *Acta2* and *Myh11* (Figure 4E,F). Ang 1-7 treatment also attenuated Ang II-induced onset of inflammatory and synthetic phenotype in abdominal aortic SMCs, resulting in reduced mRNA expressions of *Il-6, Mmp2, Mmp9, Col1a1*, and *Col3a1* (Figure 4G-K). Thus, administration of Ang 1-7 prevented Ang II-induced loss of α-SMA^+^ cells, prevented apoptosis and attenuated phenotypic switching of abdominal aortic SMCs *in vitro*, suggesting the cytoprotective role of Ang 1-7 may be involved in protection against the development of AAA in a murine model.

### Ang 1-7 reduces Ang II-induced mitochondrial fission and ROS generation in abdominal aortic SMCs

**Figure 5:**
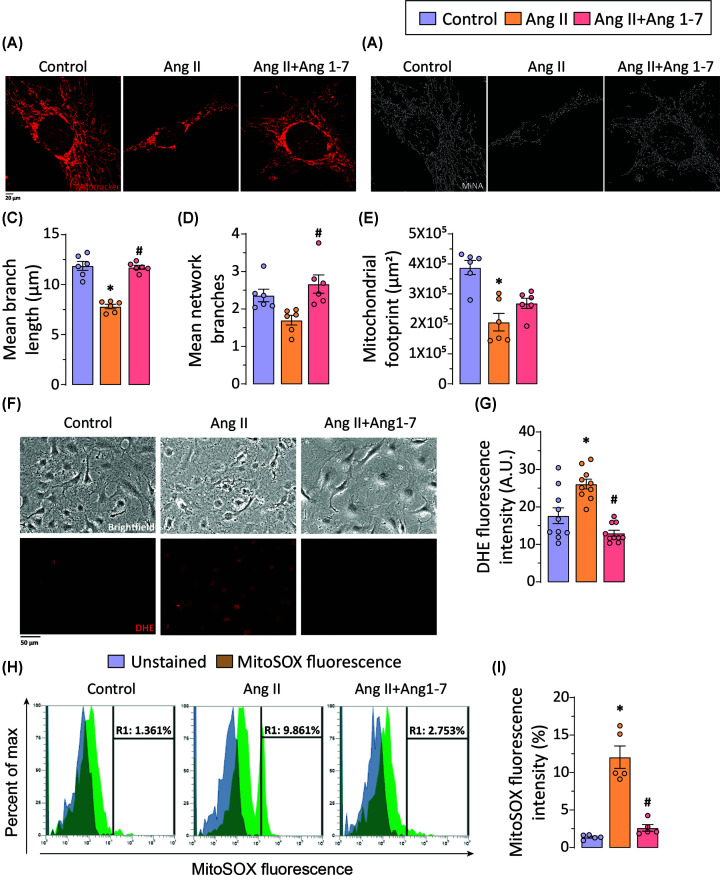
Ang II potentiates mitochondrial fission and oxidative stress in abdominal aortic VSMCs and the critical role of Ang 1-7 Representative (**A**) confocal images of Mitotracker red staining and (**B**) their analysis using the Mitochondrial Network Analysis program (MiNA plugin; ImageJ) show smaller and more fragmented mitochondria in response to Ang II-treated abdominal aortic SMCs compared with the control group. Quantification of Mitotracker red-staining images using MiNA showing decreased (**C**) mean branch length, (**D**) mean network branches, and (**E**) decreased mitochondrial footprint in Ang II-treated VSMCs compared with the control group. (A–E) Ang 1-7, treatment attenuates Ang II-induced mitochondrial fission. Representative (**F**) brightfield microscopy and confocal microscopy images of DHE-stained abdominal SMCs, along with (**G**) quantification of DHE fluorescence intensity, show increased cellular ROS levels in Ang II-treated VSMCs compared with control group. (**H**) Flow cytometric analysis of MitoSOX-stained abdominal aortic SMCs and (**I**) quantification of MitoSOX fluorescence indicate increased mitochondrial ROS levels in Ang II-treated VSMCs. (**F–I**) Ang 1-7, treatment mitigates Ang II-mediated increased cellular and mitochondrial ROS levels in abdominal aortic SMCs. Each data point on graph represents biological replicate. A.U.: Arbitrary unit; *P<0.05 compared with the control group; #*P*<0.05 compared with the Ang II group using one-way ANOVA.

*In vivo* and *in vitro* experiments indicate that pro-apoptotic properties of Ang II on abdominal aortic SMCs are crucial in AAA pathophysiology, where anti-apoptotic effects exerted by Ang 1-7 lead to the preservation of aortic structure and function. Dysregulation in mitochondrial dynamics has been linked to senescence in SMCs, predisposing them to develop cardiovascular pathologies [43,44]. To investigate the effects of Ang II and Ang 1-7 treatments on mitochondrial dynamics, we performed MitoTracker Red CMXRos staining and confocal imaging. Mitotracker Red staining of abdominal aortic SMCs revealed significantly increased mitochondrial fission in response to Ang II treatment (Figure 5A,B), leading to smaller mitochondria with shorter and lesser branches. Indeed, quantification of morphometric parameters of Mitotracker Red-stained confocal images using MiNA, an NIH ImageJ plugin, corroborated decreased mean branch length, mean network branches, and mitochondrial footprint, indicating excessive mitochondrial fragmentation in aortic SMCs treated with Ang II (Figure 5C–E). Ang 1-7 treated abdominal aortic SMCs exhibited protected architecture of mitochondrial network showing preserved mean branch length, mean network branches, and mitochondrial footprint, indicating elongated mitochondria when compared with Ang II-treated abdominal aortic SMCs. The data strongly suggested Ang 1-7-mediated inhibition of Ang II-induced mitochondrial fission in abdominal aortic SMCs.

The role of ROS-mediated protein kinase-C activation and induction of SMC apoptosis has previously been reported to be involved in the AAA pathogenesis [45], where inhibition of ROS generation has been shown to attenuate aneurysm formation [46,47]. To assess the Ang II-mediated ROS generation and the effect of Ang 1-7 on cellular and mitochondrial ROS generation, we performed dihydroethidium (DHE) and MitoSOX Red staining, respectively. For evaluation of cellular ROS generation, the DHE was used, which converts into a DNA intercalating fluorescence dye upon oxidation by ROS, which stains nuclei. Confocal imaging of DHE-stained abdominal aortic SMCs and quantification of DHE fluorescence intensity exhibited significantly elevated cellular ROS levels in Ang II-treated abdominal aortic SMCs (Figure 5F,G). MitoSOX Red mitochondrial superoxide indicator is a fluorogenic dye for highly selective detection of superoxide in the mitochondria of live cells. Staining abdominal aortic SMCs with MitoSOX Red and flow cytometric analysis showed increased mitochondrial ROS generation in Ang II-treated abdominal aortic SMCs (Figure 5H–I). Cellular and mitochondrial ROS levels were significantly reduced upon treatment with Ang 1-7, as evident in the representative confocal images and flow cytometric analysis. Our data suggest that attenuation of ROS generation and decreased apoptotic cell death may be a primary mechanism of Ang 1-7-mediated mitigation of AAA.

### Ang 1-7 abrogates apoptosis in abdominal aortic SMCs by suppressing apoptosis-associated proteins

**Figure 6:**
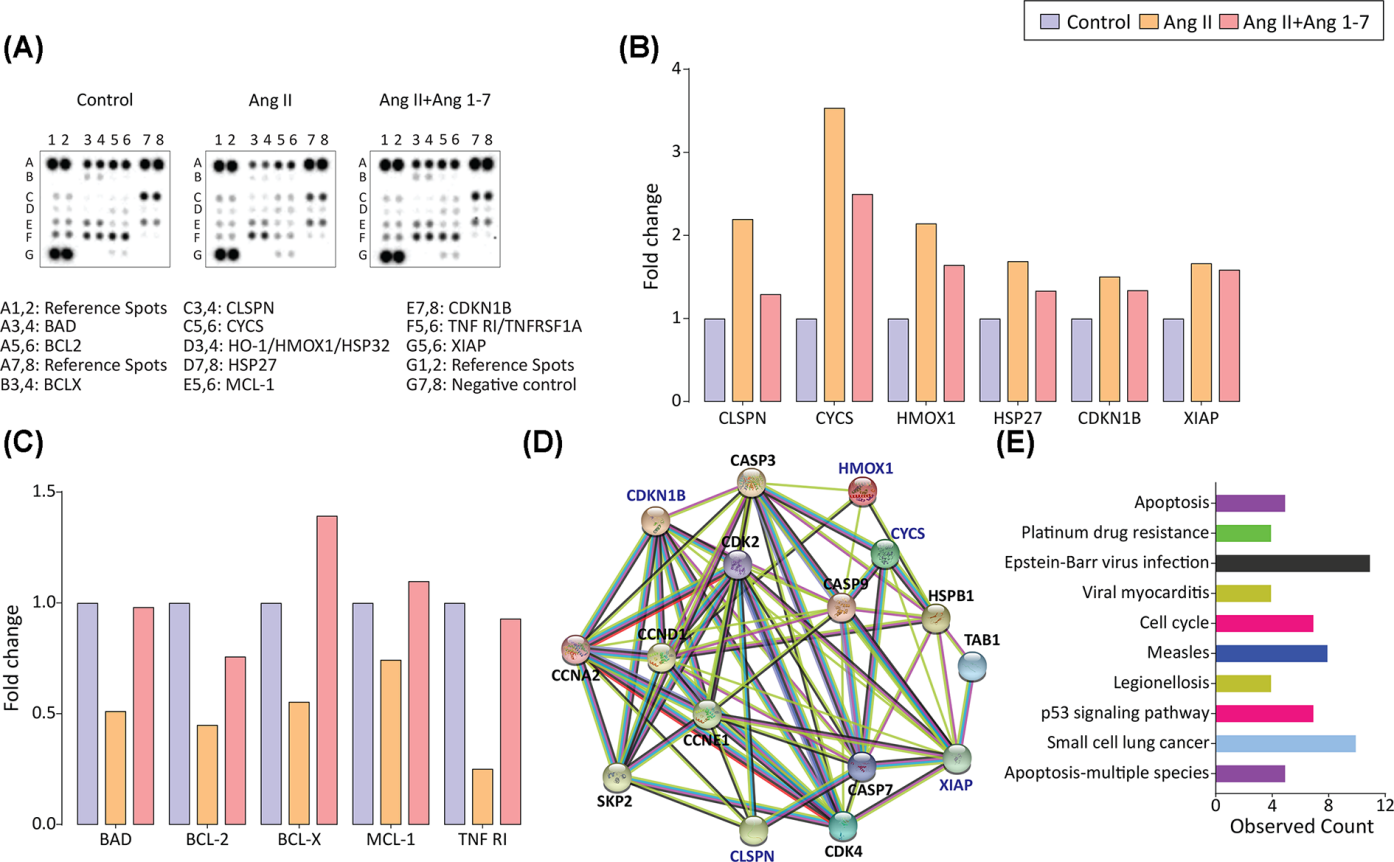
Effect of Ang II (± Ang 1-7) treatment on apoptosis-associated proteins in aortic VSMCs and functional protein–protein network analysis of differentially expressed proteins using STRING database (**A**) Protein array for assessment of apoptosis-associated protein expression in VSMCs and quantification of fold change showed (**B**) elevated and (**C**) reduced apoptosis-associated proteins in response to Ang II. Ang 1-7 attenuates VSMCs apoptosis via suppression of these regulatory proteins. Mouse apoptosis array coordinates: A1,2: Reference spots; A3,4: Bad; A5,6: Bcl-2; A7,8: Reference spots; B3,4:Bcl-x; B5,6: Caspase-3, cleaved; C1,2: Catalase; C3,4: Claspin; C5,6: Cytochrome c; C7,8: Fas/TNFRSF6/CD95; D1,2: HIF-1α; D3,4: HO-1/HMOX1/HSP32; D5,6: HO-2/HMOX2; D7,8: HSP27; E1,2: HSP60; E3,4: HSP70/HSPA1A; E5,6: MCL-1; E7,8: p27/Kip1; F1,2: p53; F3,4: SMAC/Diablo; F5,6: TNF RI/TNFRSF1A; F7,8: TRAIL R2/ TNFRSF10B; G1,2: Reference spots; G5,6: XIAP; G7,8: Negative control. (**D**) Representative images of the whole interaction network of the proteins significantly altered in Ang II(±Ang1-7) groups in the apoptosis array. Proteins identified by the apoptosis array are highlighted in blue in the protein interaction network. (**E**) KEGG functional enrichment analysis of all genes in the protein–protein interaction network revealed apoptosis and cell cycle pathways in the top 10 enriched pathways. The count is the number of genes displayed on the network and involved in each pathway.

The medial degeneration in the abdominal aorta via apoptosis of SMCs, along with ECM remodeling and ROS generation, appears to be central to the development of AAA [35,47,48]. *In vivo* and *in vitro* experiments demonstrated Ang II-induced apoptosis of abdominal aortic SMCs; Ang 1-7 attenuated apoptotic cell death. We sought to investigate the mechanisms involved in Ang 1-7-mediated cytoprotection – a proteome profiler apoptosis array kit evaluated 21 proteins critically involved in apoptosis. The apoptosis array demonstrated elevated levels of apoptosis-associated proteins in Ang II-treated abdominal aortic SMCs, including Claspin, cytochrome *c*, heme oxygenase 1 (HMOX1), Heat shock protein 27 (HSP27), p27/kip1, and X-linked inhibitor of apoptosis protein (XIAP) (Figure 6A,B). In contrast, several proteins, including BAD, BCL2, BCLX, and TNF RI, decreased in response to Ang II treatment. (Figure 6C). Ang 1-7, the treatment prevented Ang II-induced changes in the levels of various apoptosis-related proteins, including Claspin, cytochrome *c*, heme oxygenase 1 (HMOX1), BAD, BCL2, BCLX, and TNF RI (Figure 6A–C; Supplementary Figure S1). The proteins elevated in the Ang II-treated abdominal aortic SMCs were further characterized for functional interactions using the STRING database for protein-protein interactions and functional enrichment analysis (Figure 6D). Bioinformatics analyses for KEGG pathway enrichment using interactome of differentially expressed proteins in Ang II-treated abdominal aortic SMCs predicted impact apoptosis, cell cycle regulation, and the p53 signaling pathway in the abdominal aortic SMCs (Figure 6E). Our results strongly suggest that Ang II-induced mitochondrial fragmentation and increased oxidative stress led to apoptosis of abdominal aortic SMCs, which play a crucial role in developing AAA. Anti-apoptotic and antioxidant effects of Ang 1-7 play a vital role in mitigating the progression of AAA.

## Discussion

AAA is characterized by asymptomatic but progressive, irreversible dilatation of the abdominal aorta and is associated with high mortality due to the devastating consequences of aortic rupture [49]. The incidence of AAA increases with age, and its prevalence is 4-7% in males aged ≥65 years and 1–2% in females [50]. For decades, multifactorial and complex pathophysiology perplexed researchers to develop clinically efficacious pharmacological or surgical interventions. In the absence of clinically proven pharmacological treatments to prevent the progression of AAA growth or rupture, current therapeutic alternatives to prevent aortic rupture are restricted to surgical repair and endovascular intervention. Though surgical treatments have evolved into increasingly sophisticated and less invasive AAA management alternatives [51], the need to identify pathways predisposing to AAA development and shift the treatment paradigm from surgical to pharmaceutical approaches remains [15]. The precise mechanistic insight into pathogenic pathways and regulatory networks triggering aneurysmal development and subsequent dilatation is imperative for discovering targeted pharmacological agents.

The renin–angiotensin system (RAS) plays a crucial role in the regulation of mammalian physiology. Ang 1-7 is a heptapeptide generated by ACE and ACE2-mediated hydrolysis of Ang I and Ang II, respectively. Ang 1-7 exerts its actions via activation of the mas receptor, and Ang-(1-7)/ACE2/Mas axis counterbalances the vasoconstriction mediated by classical RAS. Previous studies delineated the protective effects of Ang 1-7 via the regulation of RAS [27,52,53]. Ang 1-7 has shown protective effects in cardiovascular diseases such as diabetic cardiomyopathy, atherosclerosis, hypertension, intracranial aneurysm, and heart failure due to its inherent vasodilator, anti-inflammatory, and antifibrotic properties [25,52–54]. The preclinical and clinical studies reflect the anti-inflammatory, antioxidant, and anti-fibrotic properties of Ang 1-7 in vascular disease. The role of the Ang II/AT1R axis of the RAS pathway has been widely investigated in inducing experimental AAA progression and dissection [55–57], and though Ang 1-7 is known to counterbalance Ang II actions, a detailed understanding of Ang 1-7 mediated protective effects still needs investigation. To understand mechanistic details of the effects of Ang 1-7 in AAA, we used a widely used murine model of AAA developed by Ang II administration to ApoEKO mice, which recapitulates some of the crucial features of human AAA such as marked inflammation, medial layer degeneration, and aortic rupture. However, there are certain differences observed between the murine model and human AAA. For instance, aneurysms in mice frequently occur in the suprarenal region as opposed to the infrarenal region in the human aorta. The progression of luminal dilatation in the murine model was rapid, as opposed to the gradual luminal dilatation observed in human AAA. Further, aortic dissection and rupture were observed in the murine aorta as early event in the absence of atherosclerosis. Contrarily, in humans, dissection and rupture in the abdominal aorta occur in late-stage, large aneurysms with atherosclerosis [57,58]. Considering these key differences, a translational investigation of roles of Ang 1-7 in AAA using larger animal models and humans is highly warranted. The Ang II-induced AAA exhibited severe aortic dilatation, elastin degradation, aneurysmal wall remodeling, and aortic rupture. Administration of Ang 1-7 alleviated macroscopic changes in the abdominal aorta associated with AAA development and preserved functional integrity of the aorta, evident by comparable aortic diameters and distensibility in the Ang 1-7 infused aorta. Thus, Ang 1-7 demonstrated potentially protective effects by maintaining the structural and functional integrity of the abdominal aorta.

**Figure 7:**
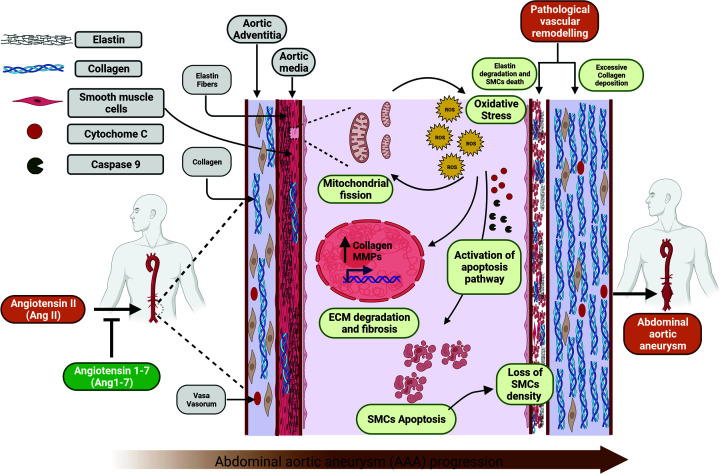
Antagonizing effects of Ang 1-7 against Ang II protects pathological alterations associated with the onset and progression of AAA Chronic infusion of Ang II results in the onset and progression of abdominal aortic aneurysm (AAA) in ApoEKO mice. Ang II triggers a pathological cascade comprising excessive mitochondrial fission, oxidative stress, vascular remodeling, and VSMCs apoptosis. The Ang II-mediated pathological mechanisms lead to the loss of structural integrity and, thus, the development of AAA. Ang 1-7 attenuates Ang II-mediated effector pathological pathways and prevents the progression of AAA. Figure created using Biorender.com.

Progression of Ang II-induced AAA is pathologically associated with excessive ECM remodeling, specifically elastic media degeneration, and profound aneurysmal wall remodeling involving perivascular collagen deposition [59]. Ang 1-7 has demonstrated anti-inflammatory, antioxidant, and anti-fibrotic properties, attenuating proteolytic activities of matrix metalloproteinases (MMPs), NADPH oxidase, and myofibroblast activation [28,60]. Our study unequivocally proves a significant decrease in elastin degradation within the aortic medial layer and a marked decrease in collagen deposition in the surrounding tissue after prolonged Ang 1-7 administration, corroborating existing literature [23]. The data corroborated Ang 1-7-mediated anti-fibrotic and inhibitory effects on adverse vascular remodeling in AAA. The progressive loss of the organized structure of the aorta wall consequently leads to weakened vessels followed by dilatation of the aorta. Although endothelial cells, fibroblasts, macrophages, and other cell types are involved in AAA development [61], a decrease in the structural integrity of the aortic vessel wall, which in part stems from the loss of medial SMCs by apoptosis and senescence appears to be central to the development of AAA [35,37]. Consistently, we observed significantly reduced SMCs density in aortic media, indicating medial degeneration. The administration of Ang 1-7 preserved the SMC population in the aortic media. The *in vitro* studies corroborated *in vivo* observations; Ang II treatment increased apoptosis of aortic SMCs isolated exclusively from the abdominal aortic region. Ang 1-7 treatment attenuated Ang II-induced apoptosis of abdominal aortic SMCs. As aortic SMCs can facilitate connective tissue repair via the production of elastin, collagen, and other matrix proteins and balancing proteolysis induced by different cell types [35], Ang 1-7-mediated preservation of SMCs in the abdominal aorta will have an important influence on the mitigation of AAA. Although apoptosis and other signaling pathways cause loss of SMCs in AAA, targeting apoptotic pathways as a potential treatment will be challenging as apoptosis has a crucial homeostatic role in maintaining vascular health (Figure 7). Thus, investigating novel therapeutic approaches must consider the unwarranted effects of apoptosis inhibition. VSMCs play a crucial role in maintaining the vascular tone in the vessel wall [62]. However, VSMCs possess a very high level of plasticity and can undergo phenotypic switch to synthetic, senescent, adipocytic, osteochondrogenic, and foam cell-like phenotypes in response to various mechanical and biochemical stimuli [63]. Research evidence points to a potential critical link between the aortic SMC phenotypic changes and the emergence of vascular remodeling and aneurysm formation [41]. The synthetic phenotype of SMCs promotes vascular inflammation and ECM remodeling, augmenting aneurysm progression [39,40]. Phenotypic switching of abdominal aortic SMCs has been identified as a predisposing factor in the development of AAA [39,40]. Our results strongly suggested that Ang II-mediated SMC phenotypic switch to synthetic phenotype promotes the progression of AAA. Importantly, Ang 1-7 attenuated pathological phenotypic switching in abdominal SMCs, reinforced the contractile phenotype, alleviated ECM remodeling, and prevented the development of AAA.

Mitochondria are dynamic organelles that alter their morphology in response to internal and external stimuli or stress through mitochondrial dynamics, fusion, and fission [64]. Defects in mitochondrial dynamics are implicated in several cardiovascular diseases; for instance, excessive mitochondrial fission has been pathogenically associated with hypertension, diabetes mellitus, and pulmonary arterial hypertension [65,66]. Increased mitochondrial fission has been associated with intrinsic and extrinsic apoptosis pathways by the induction of cytochrome c in the cytosol and apoptosis-associated caspases [67,68]. Reportedly, increased reactive oxygen species (ROS) generation and oxidative stress have resulted from abnormal mitochondrial fission and are causally associated with AAA development [69]. In our study, Ang II-treated abdominal aortic SMCs exhibited increased mitochondrial fission and oxidative stress represented by ROS generation. This corroborated the association of Ang II-induced mitochondrial fission and oxidative stress with ECM degradation and apoptotic cell death of aortic SMCs. Our observation aligns with previously reported inhibition of high glucose-induced mitochondrial fission in response to Ang 1-7 treatment of podocytes [70]. Ang 1-7 inhibited Ang II-induced mitochondrial fission and oxidative stress and attenuated apoptotic cell death of abdominal aortic SMCs. The proteome profiler apoptosis assay kit further corroborated the involvement of apoptosis-associated proteins in the Ang 1-7 mediated protection against AAA. Selective elevation of Claspin, cytochrome *c*, heme oxygenase 1 (HMOX1), Heat shock protein 27 (HSP27), p27/kip1, and X-linked inhibitor of apoptosis protein (XIAP), whereas decreased levels of BAD, BCL2, BCLX, and TNF RI in response to Ang II treatment suggests selective activation of cytochrome *c*-Caspase-9 axis being involved in the apoptosis of abdominal aortic SMCs. However, the identification of precise mechanisms requires further detailed investigations. Our results also suggest that Ang 1-7-mediated prevention of mitochondrial structure and attenuation of cytochrome *c* release prevented aortic SMC death and mitigated the progression of AAA (Figure 7).

In conclusion, based on the data reinforced with cellular and molecular investigations indicate that enhancing Ang 1-7 actions can provide a novel and promising therapeutic avenue for the treatment of AAA.

## Perspectives

- Abdominal aortic aneurysm (AAA) is a complex vascular disease associated with worldwide morbidity and mortality. Identification of cellular mechanisms and regulatory pathways involved in pathogenesis will facilitate the development of novel therapeutic agents.
- Activation of RAS, specifically Ang II, leads to pathogenic alterations, i.e., vascular remodeling, oxidative stress, and SMC apoptosis in the abdominal aorta; we aimed to study the protective effects of Ang 1-7 by antagonism of Ang II in AAA.
- Ang 1-7 exerts protective effects against Ang II-induced AAA by attenuating pathological alterations. With the unavailability of pharmacological therapeutic agents for surgical treatments with adverse side effects, management of AAA proves to be a challenging avenue. Ang 1-7-mediated protective effects in AAA indicate that enhancing Ang 1-7 actions may provide a promising therapeutic approach to the prevention of AAA.

## Supplementary Material

Supplementary Figure S1

## Data Availability

The data that support the findings of this study are available from the corresponding author upon reasonable request.

## Abbreviations

AAA: abdominal aortic aneurysm
ACE: angiotensin-converting enzyme
Ang 1-7: Angiotensin 1-7
Ang II: Angiotensin II
ECM: extracellular matrix
EKV: ECG-gated Kilohertz Visualization
MMP: matrix metalloproteinase
RAS: renin–angiotensin system
VSMC: vascular smooth muscle cell
VVG: Verhoeff-Van Gieson

## References

[1] Nordon I.M., Hinchliffe R.J., Loftus I.M. and Thompson M.M. (2011) Pathophysiology and epidemiology of abdominal aortic aneurysms. Nat. Rev. Cardiol. 8, 92–102 10.1038/nrcardio.2010.180 21079638

[2] Lattanzi S. (2020) Abdominal aortic aneurysms: pathophysiology and clinical issues. J. Intern. Med. 288, 376–378 10.1111/joim.13060 32301175

[3] Golledge J., Muller J., Daugherty A. and Norman P. (2006) Abdominal aortic aneurysm: pathogenesis and implications for management. Arterioscler. Thromb. Vasc. Biol. 26, 2605–2613 10.1161/01.ATV.0000245819.32762.cb 16973970

[4] Lu H., Rateri D.L., Bruemmer D., Cassis L.A. and Daugherty A. (2012) Involvement of the renin-angiotensin system in abdominal and thoracic aortic aneurysms. Clin. Sci. (Lond.) 123, 531–543 10.1042/CS20120097 22788237

[5] Heeneman S., Sluimer J.C. and Daemen M.J. (2007) Angiotensin-converting enzyme and vascular remodeling. Circ. Res. 101, 441–454 10.1161/CIRCRESAHA.107.148338 17761934

[6] Montezano A.C., Nguyen Dinh Cat A., Rios F.J. and Touyz R.M. (2014) Angiotensin II and vascular injury. Curr. Hypertens. Rep. 16, 431 10.1007/s11906-014-0431-2 24760441

[7] Habashi J.P., Doyle J.J., Holm T.M., Aziz H., Schoenhoff F., Bedja D. et al. (2011) Angiotensin II type 2 receptor signaling attenuates aortic aneurysm in mice through ERK antagonism. Science (New York, NY) 332, 361–365 10.1126/science.1192152 PMC3097422 21493863

[8] Kuivaniemi H., Ryer E.J., Elmore J.R. and Tromp G. (2015) Understanding the pathogenesis of abdominal aortic aneurysms. Expert Rev. Cardiovasc. Ther. 13, 975–987 10.1586/14779072.2015.1074861 26308600 PMC4829576

[9] Mastoraki S.T., Toumpoulis I.K., Anagnostopoulos C.E., Tiniakos D., Papalois A., Chamogeorgakis T.P. et al. (2012) Treatment with simvastatin inhibits the formation of abdominal aortic aneurysms in rabbits. Ann. Vasc. Surg. 26, 250–258 10.1016/j.avsg.2011.09.003 22222170

[10] Manning M.W., Cassis L.A. and Daugherty A. (2003) Differential effects of doxycycline, a broad-spectrum matrix metalloproteinase inhibitor, on angiotensin II-induced atherosclerosis and abdominal aortic aneurysms. Arterioscler. Thromb. Vasc. Biol. 23, 483–488 10.1161/01.ATV.0000058404.92759.32 12615694

[11] Xiong F., Zhao J., Zeng G., Huang B., Yuan D. and Yang Y. (2014) Inhibition of AAA in a rat model by treatment with ACEI perindopril. J. Surg. Res. 189, 166–173 10.1016/j.jss.2014.01.057 24602481

[12] Meijer C.A., Stijnen T., Wasser M.N., Hamming J.F., van Bockel J.H. and Lindeman J.H. (2013) Doxycycline for stabilization of abdominal aortic aneurysms: a randomized trial. Ann. Intern. Med. 159, 815–823 10.7326/0003-4819-159-12-201312170-00007 24490266

[13] Takagi H., Yamamoto H., Iwata K., Goto S. and Umemoto T. (2012) Effects of statin therapy on abdominal aortic aneurysm growth: a meta-analysis and meta-regression of observational comparative studies. Eur. J. Vasc. Endovasc. Surg.: Off. J. Eur. Soc. Vasc. Surg. 44, 287–292 10.1016/j.ejvs.2012.06.021 22824348

[14] Lindeman J.H. and Matsumura J.S. (2019) Pharmacologic management of aneurysms. Circ. Res. 124, 631–646 10.1161/CIRCRESAHA.118.312439 30763216 PMC6386187

[15] Chaikof E.L., Dalman R.L., Eskandari M.K., Jackson B.M., Lee W.A., Mansour M.A. et al. (2018) The society for vascular surgery practice guidelines on the care of patients with an abdominal aortic aneurysm. J. Vasc. Surg. 67, 2.e2–77.e2 10.1016/j.jvs.2017.10.044 29268916

[16] Michel M., Becquemin J.P., Marzelle J., Quelen C. and Durand-Zaleski I. (2018) Editor's Choice - A study of the cost-effectiveness of fenestrated/branched EVAR compared with open surgery for patients with complex aortic aneurysms at 2 years. Eur. J. Vasc. Endovasc. Surg.: Off. J. Eur. Soc. Vasc. Surg. 56, 15–21 10.1016/j.ejvs.2017.12.008 29342417

[17] Santos R.A., Ferreira A.J., Pinheiro S.V., Sampaio W.O., Touyz R. and Campagnole-Santos M.J. (2005) Angiotensin-(1-7) and its receptor as a potential targets for new cardiovascular drugs. Expert Opin. Investig. Drugs 14, 1019–1031 10.1517/13543784.14.8.1019 16050794

[18] Raizada M.K. and Ferreira A.J. (2007) ACE2: a new target for cardiovascular disease therapeutics. J. Cardiovasc. Pharmacol. 50, 112–119 10.1097/FJC.0b013e3180986219 17703127

[19] Trask A.J. and Ferrario C.M. (2007) Angiotensin-(1-7): pharmacology and new perspectives in cardiovascular treatments. Cardiovasc. Drug Rev. 25, 162–174 10.1111/j.1527-3466.2007.00012.x 17614938

[20] Ferrario C.M., Chappell M.C., Tallant E.A., Brosnihan K.B. and Diz D.I. (1997) Counterregulatory actions of angiotensin-(1-7). Hypertension (Dallas, Tex: 1979) 30, 535–541 10.1161/01.HYP.30.3.535 9322978

[21] Jiang F., Yang J., Zhang Y., Dong M., Wang S., Zhang Q. et al. (2014) Angiotensin-converting enzyme 2 and angiotensin 1-7: novel therapeutic targets. Nat. Rev. Cardiol. 11, 413–426 10.1038/nrcardio.2014.59 24776703 PMC7097196

[22] Shi Y., Lo C.S., Padda R., Abdo S., Chenier I., Filep J.G. et al. (2015) Angiotensin-(1-7) prevents systemic hypertension, attenuates oxidative stress and tubulointerstitial fibrosis, and normalizes renal angiotensin-converting enzyme 2 and Mas receptor expression in diabetic mice. Clin. Sci. (London, England: 1979) 128, 649–663 10.1042/CS20140329 25495544

[23] Xue F., Yang J., Cheng J., Sui W., Cheng C., Li H. et al. (2020) Angiotensin-(1-7) mitigated angiotensin II-induced abdominal aortic aneurysms in apolipoprotein E-knockout mice. Br. J. Pharmacol. 177, 1719–1734 10.1111/bph.14906 31658493 PMC7070176

[24] Patel V.B., Mori J., McLean B.A., Basu R., Das S.K., Ramprasath T. et al. (2016) ACE2 deficiency worsens epicardial adipose tissue inflammation and cardiac dysfunction in response to diet-induced obesity. Diabetes 65, 85–95 10.2337/db15-0399 26224885 PMC4686955

[25] Patel V.B., Zhong J.C., Grant M.B. and Oudit G.Y. (2016) Role of the ACE2/Angiotensin 1-7 axis of the renin-angiotensin system in heart failure. Circ. Res. 118, 1313–1326 10.1161/CIRCRESAHA.116.307708 27081112 PMC4939482

[26] Patel V.B., Zhong J.C., Fan D., Basu R., Morton J.S., Parajuli N. et al. (2014) Angiotensin-converting enzyme 2 is a critical determinant of angiotensin II-induced loss of vascular smooth muscle cells and adverse vascular remodeling. Hypertension 64, 157–164 10.1161/HYPERTENSIONAHA.114.03388 24799609

[27] Jadli A.S., Ballasy N.N., Gomes K.P., Mackay C.D.A., Meechem M., Wijesuriya T.M. et al. (2022) Attenuation of smooth muscle cell phenotypic switching by angiotensin 1-7 protects against thoracic aortic aneurysm. Int. J. Mol. Sci. 23, 1–18 10.3390/ijms232415566 36555207 PMC9779869

[28] Mori J., Patel V.B., Abo Alrob O., Basu R., Altamimi T., Desaulniers J. et al. (2014) Angiotensin 1-7 ameliorates diabetic cardiomyopathy and diastolic dysfunction in db/db mice by reducing lipotoxicity and inflammation. Circulation Heart Failure 7, 327–339 10.1161/CIRCHEARTFAILURE.113.000672 24389129

[29] Ballasy N.N., Jadli A.S., Edalat P., Kang S., Fatehi Hassanabad A., Gomes K.P. et al. (2021) Potential role of epicardial adipose tissue in coronary artery endothelial cell dysfunction in type 2 diabetes. FASEB J.: Off. Publ. Federation Am. Societies Exp. Biol. 35, e21878 10.1096/fj.202100684RR 34469050

[30] Valente A.J., Maddalena L.A., Robb E.L., Moradi F. and Stuart J.A. (2017) A simple ImageJ macro tool for analyzing mitochondrial network morphology in mammalian cell culture. Acta Histochem. 119, 315–326 10.1016/j.acthis.2017.03.001 28314612

[31] Daugherty A. and Cassis L. (2004) Angiotensin II and abdominal aortic aneurysms. Curr. Hypertens. Rep. 6, 442–446 10.1007/s11906-004-0038-0 15527688

[32] Cassis L.A., Gupte M., Thayer S., Zhang X., Charnigo R., Howatt D.A. et al. (2009) ANG II infusion promotes abdominal aortic aneurysms independent of increased blood pressure in hypercholesterolemic mice. Am. J. Physiol. Heart Circ. Physiol. 296, H1660–H1665 10.1152/ajpheart.00028.2009 19252100 PMC2685354

[33] Zha Y., Peng G., Li L., Yang C., Lu X. and Peng Z. (2017) Quantitative aortic distensibility measurement using CT in Patients with Abdominal Aortic Aneurysm: Reproducibility and Clinical Relevance. BioMed Res. Int. 2017, 5436927 10.1155/2017/5436927 28484713 PMC5412143

[34] Adams L., Brangsch J., Hamm B., Makowski M.R. and Keller S. (2021) Targeting the extracellular matrix in abdominal aortic aneurysms using molecular imaging insights. Int. J. Mol. Sci. 22, 10.3390/ijms22052685 PMC7962044 33799971

[35] López-Candales A., Holmes D.R., Liao S., Scott M.J., Wickline S.A. and Thompson R.W. (1997) Decreased vascular smooth muscle cell density in medial degeneration of human abdominal aortic aneurysms. Am. J. Pathol. 150, 993–1007 9060837 PMC1857880

[36] Zhang J., Schmidt J., Ryschich E., Schumacher H. and Allenberg J.R. (2003) Increased apoptosis and decreased density of medial smooth muscle cells in human abdominal aortic aneurysms. Chin. Med. J. 116, 1549–1552 14570621

[37] Thompson R.W., Liao S. and Curci J.A. (1997) Vascular smooth muscle cell apoptosis in abdominal aortic aneurysms. Coron. Artery Dis. 8, 623–631 10.1097/00019501-199710000-00005 9457444

[38] Li Y., Song Y.H., Mohler J. and Delafontaine P. (2006) ANG II induces apoptosis of human vascular smooth muscle via extrinsic pathway involving inhibition of Akt phosphorylation and increased FasL expression. Am. J. Physiol. Heart Circ. Physiol. 290, H2116–H2123 10.1152/ajpheart.00551.2005 16339840 PMC3217239

[39] Clément M., Chappell J., Raffort J., Lareyre F., Vandestienne M., Taylor A.L. et al. (2019) Vascular smooth muscle cell plasticity and autophagy in dissecting aortic aneurysms. Arterioscler. Thromb. Vasc. Biol. 39, 1149–1159 10.1161/ATVBAHA.118.311727 30943775 PMC6544538

[40] Wang J., Tian X., Yan C., Wu H., Bu Y., Li J. et al. (2023) TCF7L1 accelerates smooth muscle cell phenotypic switching and aggravates abdominal aortic aneurysms. JACC Basic Transl. Sci. 8, 155–170 10.1016/j.jacbts.2022.07.012 36908661 PMC9998605

[41] Alexander M.R. and Owens G.K. (2012) Epigenetic control of smooth muscle cell differentiation and phenotypic switching in vascular development and disease. Annu. Rev. Physiol. 74, 13–40 10.1146/annurev-physiol-012110-142315 22017177

[42] Malashicheva A., Kostina D., Kostina A., Irtyuga O., Voronkina I., Smagina L. et al. (2016) Phenotypic and functional changes of endothelial and smooth muscle cells in thoracic aortic aneurysms. Int. J. Vasc. Med. 2016, 3107879 10.1155/2016/3107879 26904289 PMC4745582

[43] Ma D., Zheng B., Liu H.L., Zhao Y.B., Liu X., Zhang X.H. et al. (2020) Klf5 down-regulation induces vascular senescence through eIF5a depletion and mitochondrial fission. PLoS Biol. 18, e3000808 10.1371/journal.pbio.3000808 32817651 PMC7462304

[44] Wang L., Yu T., Lee H., O'Brien D.K., Sesaki H. and Yoon Y. (2015) Decreasing mitochondrial fission diminishes vascular smooth muscle cell migration and ameliorates intimal hyperplasia. Cardiovasc. Res. 106, 272–283 10.1093/cvr/cvv005 25587046 PMC4481571

[45] Li P.F., Maasch C., Haller H., Dietz R. and von Harsdorf R. (1999) Requirement for protein kinase C in reactive oxygen species-induced apoptosis of vascular smooth muscle cells. Circulation 100, 967–973 10.1161/01.CIR.100.9.967 10468528

[46] Xiong W., Mactaggart J., Knispel R., Worth J., Zhu Z., Li Y. et al. (2009) Inhibition of reactive oxygen species attenuates aneurysm formation in a murine model. Atherosclerosis 202, 128–134 10.1016/j.atherosclerosis.2008.03.029 18502427 PMC2646364

[47] Krishna S.M., Li J., Wang Y., Moran C.S., Trollope A., Huynh P. et al. (2021) Kallistatin limits abdominal aortic aneurysm by attenuating generation of reactive oxygen species and apoptosis. Sci. Rep. 11, 17451 10.1038/s41598-021-97042-8 34465809 PMC8408144

[48] Thomas M., Gavrila D., McCormick M.L., Miller F.J. Jr, Daugherty A., Cassis L.A. et al. (2006) Deletion of p47phox attenuates angiotensin II-induced abdominal aortic aneurysm formation in apolipoprotein E-deficient mice. Circulation 114, 404–413 10.1161/CIRCULATIONAHA.105.607168 16864727 PMC3974117

[49] Daugherty A., Cassis L.A. and Lu H. (2011) Complex pathologies of angiotensin II-induced abdominal aortic aneurysms. J. Zhejiang University Sci. B. 12, 624–628 10.1631/jzus.B1101002 PMC3150714 21796801

[50] Moll F.L., Powell J.T., Fraedrich G., Verzini F., Haulon S., Waltham M. et al. (2011) Management of abdominal aortic aneurysms clinical practice guidelines of the European society for vascular surgery. Eur. J. Vasc. Endovasc. Surg.: Off. J. Eur. Soc. Vasc. Surg. 41, S1–S58 10.1016/j.ejvs.2010.09.011 21215940

[51] Greenhalgh R.M. and Powell J.T. (2008) Endovascular repair of abdominal aortic aneurysm. N. Engl. J. Med. 358, 494–501 10.1056/NEJMct0707524 18234753

[52] Zhang Y.H., Zhang Y.H., Dong X.F., Hao Q.Q., Zhou X.M., Yu Q.T. et al. (2015) ACE2 and Ang-(1-7) protect endothelial cell function and prevent early atherosclerosis by inhibiting inflammatory response. Inflammation Res.: Off. J. Eur. Histamine Res. Soc. [et al] 64, 253–260 10.1007/s00011-015-0805-1 25721616

[53] Peña Silva R.A., Kung D.K., Mitchell I.J., Alenina N., Bader M., Santos R.A. et al. (2014) Angiotensin 1-7 reduces mortality and rupture of intracranial aneurysms in mice. Hypertension (Dallas, Tex: 1979) 64, 362–368 10.1161/HYPERTENSIONAHA.114.03415 24799613 PMC4096422

[54] Zhao S., Ghosh A., Lo C.S., Chenier I., Scholey J.W., Filep J.G. et al. (2018) Nrf2 deficiency upregulates intrarenal angiotensin-converting enzyme-2 and angiotensin 1-7 receptor expression and attenuates hypertension and nephropathy in diabetic mice. Endocrinology 159, 836–852 10.1210/en.2017-00752 29211853 PMC5774246

[55] Rateri D.L., Davis F.M., Balakrishnan A., Howatt D.A., Moorleghen J.J., O'Connor W.N. et al. (2014) Angiotensin II induces region-specific medial disruption during evolution of ascending aortic aneurysms. Am. J. Pathol. 184, 2586–2595 10.1016/j.ajpath.2014.05.014 25038458 PMC4188133

[56] Trachet B., Piersigilli A., Fraga-Silva R.A., Aslanidou L., Sordet-Dessimoz J., Astolfo A. et al. (2016) Ascending aortic aneurysm in Angiotensin II-infused mice: formation, progression, and the role of focal dissections. Arterioscler. Thromb. Vasc. Biol. 36, 673–681 10.1161/ATVBAHA.116.307211 26891740

[57] Daugherty A., Manning M.W. and Cassis L.A. (2000) Angiotensin II promotes atherosclerotic lesions and aneurysms in apolipoprotein E-deficient mice. J. Clin. Invest. 105, 1605–1612 10.1172/JCI7818 10841519 PMC300846

[58] Saraff K., Babamusta F., Cassis L.A. and Daugherty A. (2003) Aortic dissection precedes formation of aneurysms and atherosclerosis in angiotensin II-infused, apolipoprotein E-deficient mice. Arterioscler. Thromb. Vasc. Biol. 23, 1621–1626 10.1161/01.ATV.0000085631.76095.64 12855482

[59] Cao R.Y., Amand T., Ford M.D., Piomelli U. and Funk C.D. (2010) The murine Angiotensin II-induced abdominal aortic aneurysm model: rupture risk and inflammatory progression patterns. Front. Pharmacol. 1, 9 10.3389/fphar.2010.00009 21713101 PMC3112241

[60] Simões e Silva A.C., Silveira K.D., Ferreira A.J. and Teixeira M.M. (2013) ACE2, angiotensin-(1-7) and Mas receptor axis in inflammation and fibrosis. Br. J. Pharmacol. 169, 477–492 10.1111/bph.12159 23488800 PMC3682698

[61] Quintana R.A. and Taylor W.R. (2019) Cellular mechanisms of aortic aneurysm formation. Circ. Res. 124, 607–618 10.1161/CIRCRESAHA.118.313187 30763207 PMC6383789

[62] Petsophonsakul P., Furmanik M., Forsythe R., Dweck M., Schurink G.W., Natour E. et al. (2019) Role of vascular smooth muscle cell phenotypic switching and calcification in aortic aneurysm formation. Arterioscler. Thromb. Vasc. Biol. 39, 1351–1368 10.1161/ATVBAHA.119.312787 31144989

[63] Sorokin V., Vickneson K., Kofidis T., Woo C.C., Lin X.Y., Foo R. et al. (2020) Role of vascular smooth muscle cell plasticity and interactions in vessel wall inflammation. Front. Immunol. 11, 599415 10.3389/fimmu.2020.599415 33324416 PMC7726011

[64] Hall A.R., Burke N., Dongworth R.K. and Hausenloy D.J. (2014) Mitochondrial fusion and fission proteins: novel therapeutic targets for combating cardiovascular disease. Br. J. Pharmacol. 171, 1890–1906 10.1111/bph.12516 24328763 PMC3976611

[65] Marsboom G., Toth P.T., Ryan J.J., Hong Z., Wu X., Fang Y.H. et al. (2012) Dynamin-related protein 1-mediated mitochondrial mitotic fission permits hyperproliferation of vascular smooth muscle cells and offers a novel therapeutic target in pulmonary hypertension. Circ. Res. 110, 1484–1497 10.1161/CIRCRESAHA.111.263848 22511751 PMC3539779

[66] Vásquez-Trincado C., García-Carvajal I., Pennanen C., Parra V., Hill J.A., Rothermel B.A. et al. (2016) Mitochondrial dynamics, mitophagy and cardiovascular disease. J. Physiol. 594, 509–525 10.1113/JP271301 26537557 PMC5341713

[67] Cheng M., Lin N., Dong D., Ma J., Su J. and Sun L. (2021) PGAM5: A crucial role in mitochondrial dynamics and programmed cell death. Eur. J. Cell Biol. 100, 151144 10.1016/j.ejcb.2020.151144 33370650

[68] Frank S., Gaume B., Bergmann-Leitner E.S., Leitner W.W., Robert E.G., Catez F. et al. (2001) The role of dynamin-related protein 1, a mediator of mitochondrial fission, in apoptosis. Dev. Cell. 1, 515–525 10.1016/S1534-5807(01)00055-7 11703942

[69] Emeto T.I., Moxon J.V., Au M. and Golledge J. (2016) Oxidative stress and abdominal aortic aneurysm: potential treatment targets. Clin. Sci.(London, England: 1979) 130, 301–315 10.1042/CS20150547 26814202

[70] Ma L., Han C., Peng T., Li N., Zhang B., Zhen X. et al. (2016) Ang-(1-7) inhibited mitochondrial fission in high-glucose-induced podocytes by upregulation of miR-30a and downregulation of Drp1 and p53. J. Chinese Med. Assoc.: JCMA 79, 597–604 10.1016/j.jcma.2016.08.006 27789249

