# Supplementary material for: Inhibition of smooth muscle cell death by Angiotensin 1-7 protects against abdominal aortic aneurysm
Source: Biosci Rep. 2023 Nov 30;43(11):BSR20230718. doi: 10.1042/BSR20230718 (PMC10695742; doi:10.1042/BSR20230718)
Supplement: Supplementary Figure S1 [file BSR-2023-0718_supp.pdf]

Inhibition of smooth muscle cell death by Angiotensin 1-7 protects against abdominal aortic aneurysm

Anshul S. Jadli[1,2], Karina P. Gomes[1,2], Noura N. Ballasy[1,2], Tishani Methsala Wijesuriya[1,2], Darrell Belke[2,3], Paul W.M. Fedak[2,3] & Vaibhav B. Patel[1,2, #]

[1]Department of Physiology and Pharmacology, Cumming School of Medicine,

[2]Libin Cardiovascular Institute,

[3]Section of Cardiac Surgery, Department of Cardiac Sciences, Cumming School of Medicine,

University of Calgary, Calgary, AB, Canada.

Running title: Ang 1-7 attenuates abdominal aortic aneurysm.

[#]Corresponding Author:

Vaibhav B. Patel, M.Pharm., Ph.D.

Department of Physiology and Pharmacology,

Cumming School of Medicine,

Libin Cardiovascular Institute,

University of Calgary,

3330 Hospital Drive NW

HMRB-G71

Calgary AB T2N 4N1

Canada.

Phone: +1(403)-220-3446

E-mail: vaibhav@ucalgary.ca; vaibhav@patellab.ca

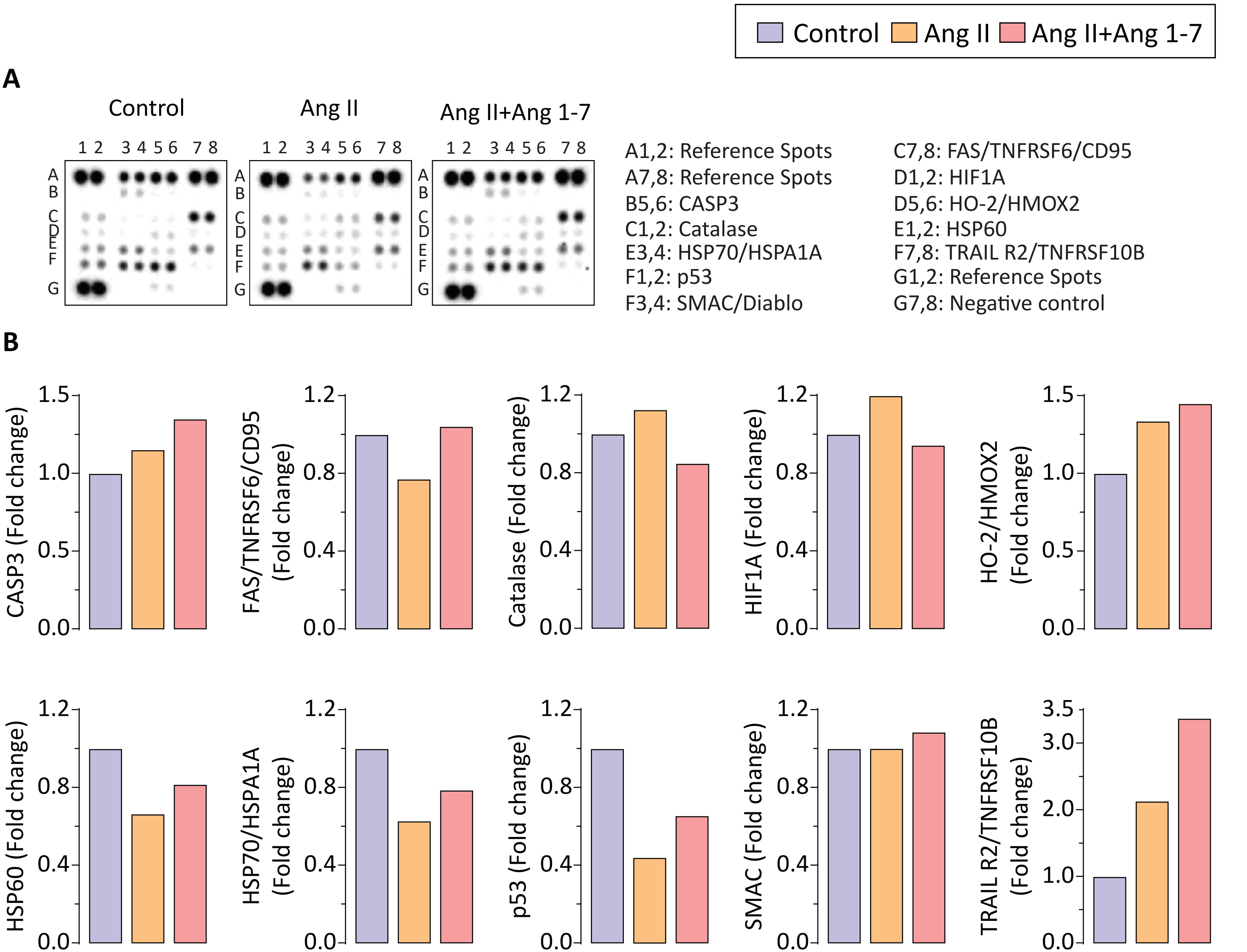

Supplementary Figure 1: Protein array for apoptosis-associated proteins. (A) Representative images for protein array blots and (B) quantification of fold change in apoptosis-associated protein expression in abdominal aortic SMCs treated with Ang II (± Ang 1-7).
